# Migrant patients in intensive care units: nursing role and cultural adaptation of humanization models — A scoping review protocol

**DOI:** 10.1016/j.mex.2026.104000

**Published:** 2026-06-10

**Authors:** Sylvain Marcel Lybrecht Llinares

**Affiliations:** a Hospital Universitario La Princesa, Madrid, Spain; b Universitat Rovira i Virgili, Tarragona, Spain

**Keywords:** Intensive care units, Emigrants and immigrants, Refugees, Cultural competency, Humanization, Nursing, Scoping review

## Abstract

Linguistic barriers in intensive care units (ICUs) are independently associated with increased mortality (OR 1.21), higher readmission rates, and reduced family participation in end-of-life decisions. Despite advances in ICU humanization, no existing model has been developed or validated for migrant or culturally and linguistically diverse populations. This protocol describes a scoping review that will map evidence on experiences of migrant patients and their families in ICUs, analyse existing humanization models and their degree of cultural adaptation, and explore the nursing role and moral distress in this context. The review follows JBI methodology and will be reported according to PRISMA-ScR guidelines. Seven bibliographic databases will be searched (PubMed/MEDLINE, CINAHL, Scopus, Web of Science, Embase, BVS, CUIDEN). The protocol is prospectively registered in OSF (DOI: https://doi.org/10.17605/OSF.IO/BE4DK).

## Highlights

• First synthesis integrating migration in critical care, ICU humanization models, and transcultural nursing within a single evidence map.
• Trilingual search strategy (English, Spanish, French) in seven databases validated against sentinel studies and registered prospectively in OSF prior to formal screening.
• Protocol addresses documented clinical inequities directly relevant to 2024 SCCM guidelines on equitable family-centred care in adult ICUs.

## Graphical abstract

**Figure:**
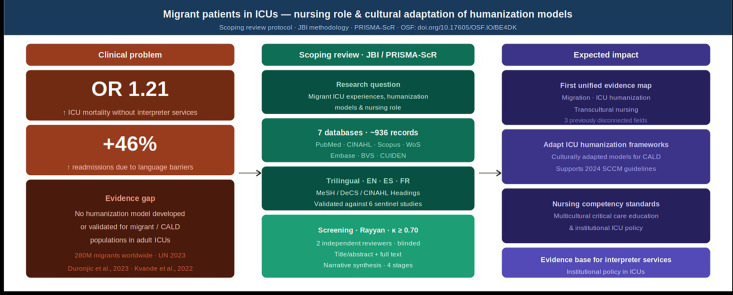

## Specifications table

**Table:** 

**Subject area**	Medicine and Dentistry
**More specific subject area**	Critical care nursing; transcultural nursing; evidence synthesis
**Name of your protocol**	Migrant patients in intensive care units: nursing role and cultural adaptation of humanization models — a scoping review protocol
**Reagents/tools**	Rayyan (Qatar Computing Research Institute) — deduplication and screening; Mendeley (Elsevier) — reference management; Microsoft Excel — data extraction and thematic coding
**Experimental design**	Scoping review following JBI methodology (Peters et al., 2024) and reported according to PRISMA-ScR guidelines (Tricco et al., 2018). Seven bibliographic databases searched; narrative synthesis with thematic grouping across five specific objectives.
**Trial registration**	Open Science Framework (OSF). Project ID: BE4DK. DOI: https://doi.org/10.17605/OSF.IO/BE4DK. Registration date: 02 May 2026 (prospective — prior to formal screening).
**Ethics**	This scoping review does not involve human participants, animal experiments, or data collected from social media platforms. It will utilise data from previously published studies. Therefore, ethical approval and informed consent are not required.
**Value of the Protocol**	• First synthesis to integrate three previously disconnected bodies of evidence: migration in critical care, ICU humanization models, and transcultural nursing perspectives.
	• Addresses documented clinical inequities: absent professional interpretation in ICUs is independently associated with increased mortality (OR 1.21) and 46% higher readmission probability.
	• Directly supports implementation of 2024 SCCM guidelines, which explicitly recommend systematic identification of equitable care barriers in adult ICUs.

## Background

In 2023, >280 million people lived outside their country of origin, making international migration a key determinant of health inequity [1] Migrant populations — including economic migrants, refugees, asylum seekers, and undocumented persons — face disproportionate barriers to healthcare access attributable to linguistic, administrative, cultural, and socioeconomic factors [2] These inequities intensify in intensive care units (ICUs), where clinical urgency, technological complexity, and high-stakes communication are paramount.

Linguistic barriers in ICUs have measurable clinical consequences. Professional interpreter use is associated with reduced ICU mortality (OR 0.81; 95% CI 0.74–0.89), while absent interpretation is associated with increased mortality (OR 1.21) and a 46% higher readmission probability [3] Families with limited language proficiency receive less information and participate less in ICU family conferences, [4,5] and end-of-life decisions are more frequently made without adequate comprehension [6] In France, undocumented migrant ICU admissions (2011–2018) showed greater severity and higher organ support requirements than the general ICU population [7].

The humanization of ICU care has consolidated as an international priority. The 2024 Society of Critical Care Medicine guidelines explicitly recommend systematic identification of equitable care barriers [8] Yet no existing humanization model has been developed or validated specifically for migrant or CALD populations in ICUs [9,10] ICU nurses report significant moral distress and unmet educational needs when caring for patients with linguistic barriers, [11,12] and no review has synthesized these nursing-specific experiences within a humanization framework.

Two recent scoping reviews address adjacent questions: Sundararajan et al. [13] examined end-of-life care for CALD patients in ICUs, and Mihu et al. [10] mapped nursing strategies for multicultural critically ill patients. Neither integrates humanization models as an analytical framework nor examines moral distress within a migration context. This protocol addresses those gaps by integrating three previously disconnected bodies of evidence: migration in critical care, ICU humanization, and transcultural nursing.

## Description of protocol

### Review questions

Primary question: What evidence exists on the experiences of migrant patients and their families in ICUs, what humanization models have been developed or applied in this context, and to what extent do these models incorporate principles of cultural competence from a nursing perspective?

Secondary questions:

What experiences, barriers, and facilitators do migrant patients and their families describe during ICU admission?
What ICU humanization models exist, and what elements of cultural adaptation do they incorporate?
What nursing interventions have been described or evaluated to improve care for migrant patients in ICUs?
What moral distress experiences do ICU nurses report when caring for patients with linguistic or cultural barriers?
What knowledge gaps exist to guide the development of culturally adapted nursing models in ICUs?

### Design and methodology

This scoping review follows the JBI methodology [14] and will be reported according to PRISMA-ScR guidelines [15,16] The choice of scoping review over systematic review reflects the emergent and heterogeneous nature of the field: no standardised interventions exist, qualitative and descriptive studies predominate, and evidence is dispersed across disciplines. A scoping review enables mapping of the scope and nature of evidence, integration of heterogeneous study types, and explicit identification of knowledge gaps. The protocol was prospectively registered in OSF prior to commencement of formal screening (Project ID: BE4DK; registration date: 02 May 2026) [17].

### Eligibility criteria (PCC framework)

Eligibility criteria follow the PCC framework (Participants, Concept, Context) as recommended by JBI methodology [14] The SPIDER framework, which informed the search strategy development, is consistent with this PCC operationalisation [18], Table 1.

**Table 1:** PCC eligibility framework.

PCC element	Operational definition
Participants	Adult migrant patients, refugees, asylum seekers, undocumented persons, and individuals from culturally and linguistically diverse (CALD) or limited English proficiency (LEP) backgrounds admitted to adult ICUs; their family members; ICU nurses caring for these populations. These categories are not mutually exclusive; they will be recorded as descriptive variables during data extraction and distinguished analytically in the synthesis.
Concept	Experiences, barriers, and facilitators of ICU care; ICU humanization models and their degree of cultural adaptation; nursing interventions for culturally and linguistically diverse patients; moral distress among ICU nurses related to linguistic and cultural barriers; communication practices and interpreter use. Humanization of care is defined as the set of practices, attitudes, and structural features that recognize patients and families as full persons, promoting dignity, communication, autonomy, and relational continuity in the ICU environment. Cultural adaptation refers to the degree to which a model or intervention explicitly integrates cultural and linguistic diversity in its design, components, or implementation guidance.
Context	Intensive care units (adult or mixed). No geographic restriction is applied. Studies from any country will be considered provided they address the review concept in an ICU setting. Country and health system context will be recorded as a variable during data extraction to enable contextual analysis of transferability.

Any study design will be considered, including qualitative, quantitative, mixed-methods, systematic or scoping reviews, clinical guidelines, position papers, and grey literature. No date restriction will be applied. Publications in English, Spanish, and French will be included. Studies conducted exclusively in paediatric or neonatal ICUs will be excluded.

### Search strategy

A systematic search will be conducted across seven bibliographic databases: PubMed/MEDLINE (PubMed interface), CINAHL (EBSCOhost), Scopus (Elsevier), Web of Science Core Collection (Clarivate), Embase (Ovid), BVS/Virtual Health Library covering LILACS, IBECS, and BDENF (BIREME interface), and CUIDEN (Fundación Index). Grey literature will be searched in institutional repositories (WHO, ECDC, SCCM, SEEIUC, SFAR) and Google Scholar (first 200 results). Reference management will be conducted using Mendeley.

The strategy combines three conceptual blocks — Participants (Block 1), Context (Block 2), and Concept (Block 3) — using Boolean operators AND/OR, with controlled vocabulary (MeSH, DeCS, CINAHL Headings, Emtree) and free-text terms in English, Spanish, and French, adapted for each database. Neonatal and paediatric ICU terms are explicitly excluded from Block 2. The strategy was validated against six sentinel studies known a priori to be relevant [3,4,9,10,13] — all were retrieved by the combined search, confirming adequate sensitivity.

**Table:** 

#	Search string
1	MeSH: "Emigrants and Immigrants"[MeSH] OR "Refugees"[MeSH] OR "Undocumented Immigrants"[MeSH] OR "Communication Barriers"[MeSH]
2	Free text (participants): migrant*[tiab] OR refugee*[tiab] OR "asylum seeker*"[tiab] OR undocumented[tiab] OR CALD[tiab] OR "limited English proficiency"[tiab] OR LEP[tiab] OR "language barrier*"[tiab] OR "cultural barrier*"[tiab] OR "ethnic minorit*"[tiab] OR "foreign born"[tiab] OR "foreign-born"[tiab]

Block 1	#1 OR #2
3	MeSH (context): "Intensive Care Units"[MeSH] OR "Critical Care"[MeSH] OR "Critical Care Nursing"[MeSH]
4	Free text (context): "intensive care unit*"[tiab] OR ICU[tiab] OR ICUs[tiab] OR "critical care"[tiab] OR "critically ill"[tiab] OR "intensive care nurs*"[tiab]
5	Exclusions: "Intensive Care Units, Neonatal"[MeSH] OR "Intensive Care Units, Pediatric"[MeSH] OR NICU[tiab] OR PICU[tiab] OR "neonatal intensive"[tiab] OR "pediatric intensive"[tiab] OR "paediatric intensive"[tiab]

Block 2	(#3 OR #4) NOT #5
6	MeSH (concept): "Humanism"[MeSH] OR "Patient-Centered Care"[MeSH] OR "Family Nursing"[MeSH] OR "Cultural Competency"[MeSH] OR "Culturally Competent Care"[MeSH] OR "Nurse-Patient Relations"[MeSH] OR "Cross-Cultural Communication"[MeSH] OR "Burnout, Professional"[MeSH]
7	Free text (concept): humaniz*[tiab] OR humanis*[tiab] OR "patient-centered"[tiab] OR "person-centered"[tiab] OR "family-centered"[tiab] OR "cultural competenc*"[tiab] OR "culturally competent care"[tiab] OR "cultural safety"[tiab] OR "cultural humility"[tiab] OR "transcultural nurs*"[tiab] OR "moral distress"[tiab] OR "moral injury"[tiab] OR interpreter*[tiab] OR "health equity"[tiab] OR "health disparit*"[tiab]

Block 3	#6 OR #7
FINAL	Block 1 AND Block 2 AND Block 3 → 139 records (02 May 2026)

Search strategies for the remaining six databases (CINAHL, Scopus, Web of Science, Embase, BVS/DeCS, CUIDEN) are provided in Supplementary Material (Appendix I–VI), available online. All strategies follow the same three-block structure adapted to each database's controlled vocabulary and interface syntax.

**Table:** 

Database	Interface	Date	Records (n)	Est. unique
PubMed/MEDLINE	PubMed	02 May 2026	139	139
CINAHL	EBSCOhost	02 May 2026	178	∼120
Scopus	Elsevier Scopus	02 May 2026	168	∼80
Web of Science	Clarivate WoS	02 May 2026	93	∼50
BVS (LILACS, IBECS, BDENF)	BIREME/BVS	02 May 2026	355	∼35
CUIDEN	Fundación Index	02 May 2026	3	∼1
Embase	Ovid	Pending	—	∼150*
**Total**			**936 + Embase**	**∼420–460**

*Note.* *Embase estimate based on 70–80% overlap with PubMed and Scopus in this subject area; actual count will update the OSF registration prior to formal screening. After full deduplication, an estimated 420–460 unique records are anticipated.

### Source of evidence selection

All records will be imported into Rayyan (Qatar Computing Research Institute) for deduplication and screening. Two independent reviewers will screen all titles and abstracts (Phase 1) with blinding of authors, journal name, year, and institutional affiliation. Full-text records will be assessed for eligibility (Phase 2) with reasons for exclusion documented using standardised categories. Discrepancies will be resolved by consensus; persistent disagreements will be adjudicated by a third reviewer. Inter-rater agreement will be assessed using Cohen's kappa (threshold: κ ≥ 0.70). A pilot screening of 25 randomly selected records will calibrate reviewer decisions before full screening. Results will be reported in a PRISMA-ScR flow diagram.

### Data extraction

Data will be extracted using a standardised instrument piloted on the first 5 included studies, performed independently by 2 reviewers with discrepancies resolved by consensus or a third reviewer Table 2.

**Table 2:** Data extraction instrument.

Category	Variables to extract
Metadata	Author(s), year, country, health system context, journal or source type, language, study design, funding source, conflicts of interest.
Population	Migrant subgroup(s) (refugee, asylum seeker, undocumented, CALD, LEP); family members or informal caregivers; ICU nurses or multidisciplinary team; sample size; ICU type (medical, surgical, mixed, trauma).
Patient/family experiences	Reported experiences of ICU care; barriers and facilitators to communication, participation, and decision-making; perceptions of dignity, information, and humanization.
Humanization models	Model or framework described, applied, or evaluated; components related to communication, dignity, family involvement, or cultural diversity; explicit cultural adaptation elements (yes/no/partial).
Nursing interventions	Specific nursing interventions described or evaluated; cultural competence strategies; interpreter use practices; educational approaches.
Moral distress	Descriptions of moral distress, moral injury, or ethical conflict among ICU nurses related to linguistic or cultural barriers; triggers identified; coping strategies reported.
Qualitative synthesis variables	Key themes; representative quotes (qualitative studies); authors' interpretive conclusions.
Evidence gaps	Areas identified by authors as requiring further research; unanswered questions.

For mixed-methods studies, qualitative and quantitative data will be extracted separately and integrated at the synthesis stage. French-language studies will be extracted by the principal investigator (native French proficiency); all extracted data will be recorded in English.

### Data synthesis

Analysis will follow a narrative synthesis approach with thematic grouping, consistent with JBI recommendations [14] No meta-analysis or formal quality appraisal will be conducted. Synthesis will proceed in four stages: (1) descriptive mapping of study characteristics; (2) thematic synthesis across the five specific objectives; (3) evidence gap mapping; and (4) integration of findings into a conceptual summary. Results will be presented in tabular and narrative form with a PRISMA-ScR flow diagram.

### Protocol validation

Prospective registration in OSF (DOI: https://doi.org/10.17605/OSF.IO/BE4DK) prior to formal screening provides methodological transparency and reduces post-hoc modifications to eligibility criteria. The search strategy was validated against six sentinel studies known a priori to be relevant [3,4,9,10,13] — all were retrieved by the combined search across all seven databases, confirming adequate sensitivity. The strategy was additionally reviewed by a second investigator with experience in evidence synthesis. The PCC and SPIDER frameworks were independently operationalised and reconciled, confirming consistency. These validation steps confirm the reproducibility and rigour of the protocol prior to execution.

## Limitations

• Restriction to English, Spanish, and French may exclude studies in Arabic, Mandarin, or other languages spoken by populations of interest; this will be documented as a limitation in the final review.
• The research team is small and the protocol was developed by a single author, which limits the independence of dual screening and extraction; however, two methodological collaborators will participate in the screening and extraction phases, and inter-rater agreement will be formally assessed (κ ≥ 0.70).
• Grey literature searches, while systematic, may not achieve the same reproducibility as database searches.
• The investigator's clinical context (adult ICU in Spain) and binational (Franco-Spanish) professional perspective may influence interpretation of findings; this is acknowledged as a reflexivity consideration.

## Author instructions

Before you complete this template, a few important points to note:

• This template is for a protocol article, for example, designed for a clinical or drug trial. If you want to describe a method or changes you’ve made to a method, please complete our method article template.
• The format of a protocol article is very different from a traditional research article. To help you write yours, we have created this template. We will only consider protocol articles submitted using this template.
• Please consult the MethodsX Guide for Authors; it highlights mandatory requirements and is packed with useful advice. We have also developed a step-by-step video guide to completing this template, designed to maximize your chances of acceptance.
• Brief instructions are in blue italics. Additional instructions appear in comment boxes to the right of the main text.

Please do not change any section headings (e.g., *Article information, Specifications table*).

## Still have questions?

➢ Email our editorial team at mex-me@elsevier.com.

Now you are ready to fill in the template below. As you complete each section, it’s important to read the associated instructions carefully. All sections are mandatory.

## CRediT author statement

SMLL: Conceptualization; Methodology; Data curation (search strategy development and execution); Writing – original draft; Writing – review and editing; Validation; Project administration.

The protocol was developed by a single author. Two methodological collaborators will participate in the screening and data extraction phases of the review execution, acting as independent reviewers; their contribution is limited to these phases and does not constitute authorship of this protocol. They are expected to be included as co-authors of the final scoping review report. This limitation is acknowledged in the Limitations section.

## Related research article

None.

## Declaration of competing interest

The authors declare that they have no known competing financial interests or personal relationships that could have appeared to influence the work reported in this paper.

## Data Availability

No data was used for the research described in the article.

## Appendix Supplementary materials

Image, application 1

## References

[1] United Nations, Department of Economic and Social Affairs, Population Division. International migration 2023: Highlights. New York: United Nations; 2023. Available from: https://www.un.org/development/desa/pd/.

[2] Lebano A. (2020). Migrants' and refugees' health status and healthcare in Europe: a scoping literature review. BMC Public Health.

[3] Duronjic A. (2023). The impact of language barriers and interpreters on critical care patient outcomes. J. Crit. Care.

[4] Espinoza Suarez N.R. (2021). Consequences of suboptimal communication for patients with limited English proficiency in the ICU. J. Crit. Care.

[5] Thornton J.D. (2009). Families with limited English proficiency receive less information in interpreted ICU family conferences. Crit. Care Med..

[6] Barwise A.K. (2019). End-of-life decision-making for ICU patients with limited English proficiency: a qualitative study. Crit. Care Med..

[7] Hraiech S. (2022). Undocumented migrants in French ICUs 2011–2018: a retrospective nationwide study. Intensive Care Med..

[8] Hwang D.Y. (2025). SCCM guidelines on family-centered care for adult ICUs: 2024. Crit. Care Med..

[9] Kvande M.E., Angel S., Nielsen A.H. (2022). Humanizing intensive care: a scoping review (HumanIC). Nurs. Ethics.

[10] Mihu L., Marques R.M.D. (2024). Pontifice Sousa P. Strategies for nursing care of critically ill multicultural patients: a scoping review. J. Clin. Nurs..

[11] Watson A.L. (2026). When words fail: ICU nurses' experiences caring for patients with limited English proficiency. J. Adv. Nurs..

[12] Gutysz-Wojnicka A. (2022). Educational needs of European ICU nurses with respect to multicultural care. Int. J. Environ. Res. Public Health.

[13] Sundararajan K. (2024). Insights from CALD patients and families about end-of-life care in the ICU: a scoping review. BMJ Open.

[14] Peters MDJ, Godfrey C, McInerney P, Munn Z, Tricco AC, Khalil H. Chapter 11: Scoping reviews. In: Aromataris E, Munn Z, editors. JBI Manual for Evidence Synthesis. Adelaide: JBI; 2024. Available from: https://jbi-global-wiki.refined.site/space/MANUAL.

[15] Tricco A.C. (2018). PRISMA extension for scoping reviews (PRISMA-ScR). Ann. Intern. Med..

[16] Peters M.D.J. (2022). Best practice guidance for scoping review protocols. JBI Evid. Synth..

[17] SM. Lybrecht‑Llinares OSF protocol registration 2026 10.17605/OSF.IO/BE4DK.

[18] Cooke A., Smith D., Booth A. (2012). Beyond PICO: the SPIDER tool for qualitative evidence synthesis. Qual. Health Res..

