# Supplementary material for: Migrant patients in intensive care units: nursing role and cultural adaptation of humanization models — A scoping review protocol
Source: MethodsX. 2026 Jun 10;17:104000. doi: 10.1016/j.mex.2026.104000 (PMC13292647; doi:10.1016/j.mex.2026.104000)
Supplement: Supplementary file 1 [file mmc1.docx]

**Supplementary material – Complete search strategies**

***Search strings***

**PubMed/MEDLINE**

*Block 1 — Participants:*

("Emigrants and Immigrants"[MeSH] OR "Refugees"[MeSH] OR "Undocumented Immigrants"[MeSH] OR "Communication Barriers"[MeSH] OR "migrant*"[tiab] OR "refugee*"[tiab] OR "asylum seeker*"[tiab] OR "undocumented"[tiab] OR "CALD"[tiab] OR "limited English proficiency"[tiab] OR "LEP"[tiab] OR "language barrier*"[tiab] OR "cultural barrier*"[tiab] OR "ethnic minorit*"[tiab] OR "foreign born"[tiab] OR "foreign-born"[tiab])

*Block 2 — Context:*

(("Intensive Care Units"[MeSH] OR "Critical Care"[MeSH] OR "Critical Care Nursing"[MeSH] OR "intensive care unit*"[tiab] OR "ICU"[tiab] OR "ICUs"[tiab] OR "critical care"[tiab] OR "critically ill"[tiab] OR "intensive care nurs*"[tiab]) NOT ("Intensive Care Units, Neonatal"[MeSH] OR "Intensive Care Units, Pediatric"[MeSH] OR "NICU"[tiab] OR "PICU"[tiab] OR "neonatal intensive"[tiab] OR "pediatric intensive"[tiab] OR "paediatric intensive"[tiab]))

*Block 3 — Concept:*

("Humanism"[MeSH] OR "Patient-Centered Care"[MeSH] OR "Family Nursing"[MeSH] OR "Cultural Competency"[MeSH] OR "Culturally Competent Care"[MeSH] OR "Nurse-Patient Relations"[MeSH] OR "Cross-Cultural Communication"[MeSH] OR "Burnout, Professional"[MeSH] OR "humaniz*"[tiab] OR "humanis*"[tiab] OR "patient-centered"[tiab] OR "person-centered"[tiab] OR "family-centered"[tiab] OR "cultural competenc*"[tiab] OR "culturally competent care"[tiab] OR "cultural safety"[tiab] OR "cultural humility"[tiab] OR "transcultural nurs*"[tiab] OR "moral distress"[tiab] OR "moral injury"[tiab] OR "interpreter*"[tiab] OR "health equity"[tiab] OR "health disparit*"[tiab])

*Combined:*

Block 1 AND Block 2 AND Block 3

**CINAHL (EBSCOhost)**

*Block 1 — Participants:*

(MH "Emigrants and Immigrants" OR MH "Refugees" OR MH "Communication Barriers" OR MH "Health Equity" OR TI migrant* OR AB migrant* OR TI refugee* OR AB refugee* OR TI "asylum seeker*" OR AB "asylum seeker*" OR TI "language barrier*" OR AB "language barrier*" OR TI "limited English proficiency" OR AB "limited English proficiency" OR TI CALD OR AB CALD OR TI "cultural barrier*" OR AB "cultural barrier*" OR TI "foreign-born" OR AB "foreign-born")

*Block 2 — Context:*

(MH "Intensive Care Units" OR MH "Critical Care" OR MH "Critical Care Nursing" OR TI "intensive care" OR AB "intensive care" OR TI ICU OR AB ICU OR TI ICUs OR AB ICUs OR TI "critical care" OR AB "critical care") NOT (MH "Intensive Care Units, Neonatal" OR MH "Intensive Care Units, Pediatric" OR TI neonatal OR TI pediatric OR TI paediatric OR TI "NICU" OR TI "PICU")

*Block 3 — Concept:*

(MH "Culturally Competent Care" OR MH "Cultural Humility" OR MH "Moral Distress" OR MH "Humanistic Nursing" OR MH "Patient-Centered Care" OR MH "Family-Centered Care" OR MH "Communication Barriers" OR TI humaniz* OR AB humaniz* OR TI humanis* OR AB humanis* OR TI "cultural competenc*" OR AB "cultural competenc*" OR TI "culturally competent care" OR AB "culturally competent care" OR TI "cultural safety" OR AB "cultural safety" OR TI "moral distress" OR AB "moral distress" OR TI interpreter* OR AB interpreter* OR TI "transcultural nurs*" OR AB "transcultural nurs*" OR TI "health disparit*" OR AB "health disparit*")

*Combined:*

Block 1 AND Block 2 AND Block 3

**Scopus**

*Block 1 — Participants:*

TITLE-ABS-KEY(migrant* OR refugee* OR "asylum seeker*" OR "undocumented" OR "language barrier*" OR "communication barrier*" OR "limited English proficiency" OR "CALD" OR "LEP" OR "culturally diverse" OR "ethnic minorit*" OR "foreign born" OR "foreign-born")

*Block 2 — Context:*

TITLE-ABS-KEY("intensive care unit*" OR "ICU" OR "ICUs" OR "critical care" OR "critically ill" OR "intensive care nurs*") NOT TITLE-ABS-KEY("neonatal intensive" OR "pediatric intensive" OR "paediatric intensive" OR "NICU" OR "PICU")

*Block 3 — Concept:*

TITLE-ABS-KEY(humaniz* OR humanis* OR "patient-centered care" OR "person-centered care" OR "family-centered care" OR "cultural competenc*" OR "culturally competent care" OR "cultural safety" OR "cultural humility" OR "transcultural nurs*" OR "moral distress" OR interpreter* OR "health equity" OR "health disparit*")

*Combined:*

Block 1 AND Block 2 AND Block 3

**Web of Science**

*Block 1 — Participants:*

TS=(migrant* OR refugee* OR "asylum seeker*" OR "language barrier*" OR "communication barrier*" OR "limited English proficiency" OR CALD OR LEP OR "culturally diverse" OR "ethnic minorit*" OR "foreign-born")

*Block 2 — Context:*

TS=("intensive care unit*" OR ICU OR "critical care" OR "critically ill" OR "intensive care nurs*") NOT TS=("neonatal intensive" OR "pediatric intensive" OR "paediatric intensive" OR NICU OR PICU)

*Block 3 — Concept:*

TS=(humaniz* OR humanis* OR "patient-centered care" OR "person-centered care" OR "cultural competenc*" OR "culturally competent care" OR "cultural safety" OR "cultural humility" OR "transcultural nurs*" OR "moral distress" OR interpreter* OR "health equity" OR "health disparit*")

*Combined:*

Block 1 AND Block 2 AND Block 3

**IBECS (BVS/DeCS)**

*Block 1 — Participants:*

("Emigrantes e Inmigrantes"[DeCS] OR "Refugiados"[DeCS] OR "Inmigrantes Indocumentados"[DeCS] OR "Barreras de Comunicación"[DeCS] OR "Equidad en Salud"[DeCS] OR migrante*[tiab] OR refugiado*[tiab] OR "barreras lingüísticas"[tiab] OR "diversidad cultural"[tiab] OR "minorías étnicas"[tiab] OR "competencia cultural"[tiab])

*Block 2 — Context:*

("Unidades de Cuidados Intensivos"[DeCS] OR "Cuidados Críticos"[DeCS] OR "Enfermería de Cuidados Críticos"[DeCS] OR "unidad de cuidados intensivos"[tiab] OR UCI[tiab])

*Block 3 — Concept:*

("Humanización de la Atención"[DeCS] OR "Atención Centrada en el Paciente"[DeCS] OR "Competencia Cultural"[DeCS] OR "Comunicación Intercultural"[DeCS] OR humanizaci*[tiab] OR "competencia cultural"[tiab] OR "seguridad cultural"[tiab] OR "angustia moral"[tiab] OR intérprete*[tiab] OR "equidad en salud"[tiab] OR "desigualdades en salud"[tiab])

*Combined:*

Block 1 AND Block 2 AND Block 3

**CUIDEN**

(migrante* OR refugiado* OR "barreras lingüísticas") AND ("cuidados intensivos" OR UCI)

**EMBASE (Ovid)**

*Block 1 — Participants:*

exp emigrant/ OR exp refugee/ OR exp illegal immigrant/ OR migrant*.tw OR refugee*.tw OR “asylum seeker*”.tw OR CALD.tw OR “limited English proficiency”.tw OR “language barrier*”.tw OR “cultural barrier*”.tw OR “ethnic minorit*”.tw OR “foreign born”.tw

*Block 2 — Context:*

(exp intensive care unit/ OR exp critical care/ OR “intensive care unit*”.tw OR ICU.tw OR “critical care”.tw OR “critically ill”.tw) NOT (exp neonatal intensive care unit/ OR exp pediatric intensive care unit/ OR NICU.tw OR PICU.tw OR “neonatal intensive”.tw OR “paediatric intensive”.tw)

*Block 3 — Concept:*

exp humanization of care/ OR exp patient centred care/ OR exp cultural competence/ OR exp moral distress/ OR exp nurse patient relationship/ OR humaniz*.tw OR humanis*.tw OR “cultural competenc*”.tw OR “culturally competent care”.tw OR “cultural safety”.tw OR “cultural humility”.tw OR “transcultural nurs*”.tw OR “moral distress”.tw OR interpreter*.tw OR “health equity”.tw OR “health disparit*”.tw

*Combined:*

Block 1 AND Block 2 AND Block 3

**Estimated ~150 records based on 70–80% overlap with PubMed+Scopus. Actual count to be updated in OSF registration prior to formal screening.*
